# Supplementary material for: A generalised significance test for individual communities in networks
Source: Sci Rep. 2018 May 9;8:7351. doi: 10.1038/s41598-018-25560-z (PMC5943579; doi:10.1038/s41598-018-25560-z)
Supplement: Supplementary file 1 — Supplementary Information [file 41598_2018_25560_MOESM1_ESM.pdf]

Supplementary Information: A generalised significance test for individual communities in networks

Sadamori Kojaku and Naoki Masuda

I. p-VALUE UNDER THE NULL MODEL

In our statistical test, the p-value is given by

$$F_{\tilde{q}}(q_c) = \int_{q_c}^{\infty} P(\tilde{q}|s_c)\mathrm{d}\tilde{q}, \quad (1)$$

where q_c is the quality of a focal community c, $\tilde{q}$ is the quality of a community detected in the randomised networks and s_c is the size of the focal community. Function $F_{\tilde{q}}(q_c)$ is the cumulative probability density of $\tilde{q}$ over $[q_c, \infty]$. In general, any cumulative probability density $F_X(Y)$ for continuous variables X and Y obeys a uniform distribution over $[0, 1]$ if Y obeys the same probability distribution as that of X [1]. Therefore, under the null model, where q_c and $\tilde{q}$ obey the same probability distribution, the p-value obeys the uniform distribution over $[0, 1]$.

II. DEPENDENCE OF THE STATISTICAL RESULTS ON THE NUMBER OF RANDOMISED NETWORKS

In this section, we examine the robustness of the statistical results with respect to the number of generated randomised networks. We use the 12 empirical networks used in the main text, which consist of different numbers of nodes and communities. For each community c, we compute the p-value using R randomised networks, denoted by $p_c^{[R]}$. In the main text, we set $R = 500$. Then, we generate another R randomised networks and compute the p-value, $p_c^{[2R]}$, for each community c. We measure the quality and the size of a community using q_c^{mod} and vol_c, respectively. We use the Louvain algorithm to detect communities in the randomised networks.

The p-value computed with 500 randomised networks is close to that computed with $1,000$ randomised networks for most communities (Fig. S1(a)). The Pearson correlation coefficient, denoted by r, between the p-value between 500 networks and that with $1,000$

networks is equal to $r = 0.999$. Additionally, the p-value with $R = 1,000$ is smaller than that with $R = 500$ for most communities, which indicates that the present statistical test is conservative when R is small. Therefore, with $R = 500$ employed in the main text, which is relatively small, we are not overestimating the significance of the detected communities.

A large network and community may require many samples of randomised networks, R, for a reliable estimation of the p-value. To examine this possibility, we plot the variation in the p-value, defined by $|p_c^{[R]} - p_c^{[2R]}|$, for each community c in Fig. S1(c). The variation of p-value tends to be small for large communities although the correlation between the variation and vol_c is small ($r = -0.144$). A negative (albeit weak) correlation that we have found implies that a larger community requires a smaller value of R, which encourages the application of our statistical test to networks larger than those examined in the present article. Finally, we examine the robustness of the p-value with respect to the number of nodes in the network. To this end, for each empirical network, we average the variation, $|p_c^{[R]} - p_c^{[2R]}|$, over all communities in the network. The averaged variation is not strongly correlated with the number of nodes in the networks (Fig. S1(e); $r = 0.087$).

The results remain qualitatively the same when $R = 5,000$ (Figs. S1(b), S1(d) and S1(f)).

[1] P. Embrechts and M. Hofert, Math. Meth. Ope. Res. **77**, 423 (2013).

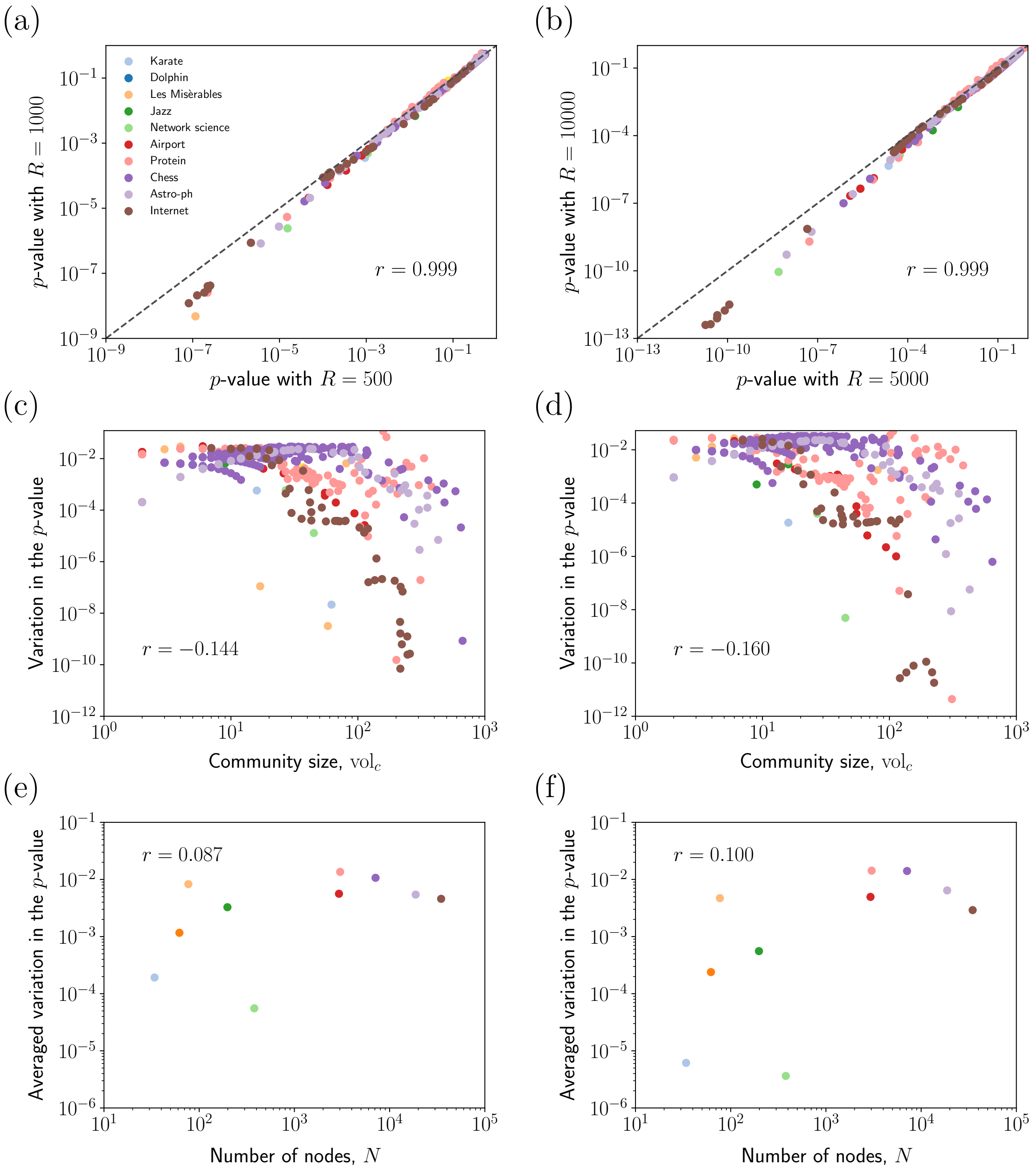

FIG. 1: Variation in the p-value computed with different numbers of randomised networks. Panels (a), (c) and (e) compare the results between $R = 500$ and $R = 1,000$. Panels (b), (d) and (f) compare the results between $R = 5,000$ and $R = 10,000$. In panels (a)–(d), each circle indicates a community detected in the original network. In panels (e) and (f), each circle indicates a network. We do not show the results for the Email and Blog networks because the p-value and their variation are less than 10^{-308}. In each panel, r denotes the Pearson correlation coefficient.
